# A Pilot Feasibility Study of Neurodevelopmental Surveillance After the Fontan Operation Using a Sedation-Free Brain MRI Approach

**DOI:** 10.3390/jcm15083069

**Published:** 2026-04-17

**Authors:** Kwang Ho Choi, Hye Jin Baek, Hyungtae Kim, Si-Chan Sung, Joung-Hee Byun, Hoon Ko, Hyoung-Doo Lee, Ra Yu Yun, Jun-Ho Kim, Stefan Skare

**Affiliations:** 1 Department of Thoracic and Cardiovascular Surgery, Research Institute for Convergence of Biomedical Science and Technology, Pusan National University Yangsan Hospital, Pusan National University School of Medicine, 20 Geumo-ro, Mulgeum-eup, Yangsan-si 50612, Republic of Korea; 2 Department of Radiology, Research Institute for Convergence of Biomedical Science and Technology, Pusan National University Yangsan Hospital, Pusan National University School of Medicine, 20 Geumo-ro, Mulgeum-eup, Yangsan-si 50612, Republic of Korea; 3 Department of Pediatrics, Research Institute for Convergence of Biomedical Science and Technology, Pusan National University Yangsan Hospital, Pusan National University School of Medicine, 20 Geumo-ro, Mulgeum-eup, Yangsan-si 50612, Republic of Korea; 4 Department of Rehabilitation Medicine, Busan Veterans Hospital, 420 Baegyang-daero, Sasang-gu, Busan 46996, Republic of Korea; 5 Department of Electrical and Electronic Engineering, Yonsei University, Seoul 03722, Republic of Korea; 6 Department of Neuroradiology, Karolinska University Hospital, 171 76 Stockholm, Sweden; 7 Department of Clinical Neuroscience, Karolinska Institute, 171 77 Stockholm, Sweden

**Keywords:** Fontan procedure, pediatric neuroimaging, ultrafast MRI, brain volumetry, sedation-free MRI, deep learning reconstruction, neurocognitive outcome

## Abstract

**Background and Objectives:** After undergoing a Fontan operation, children with single-ventricle physiology are at a risk of neurodevelopmental impairment; data from the Korean population are scarce. We characterized the neurocognitive profiles of early school-aged Fontan patients and evaluated the feasibility of a sedation-free ultrafast brain magnetic resonance imaging (MRI) protocol for volumetric analysis. **Methods:** This prospective study screened 25 children who had undergone Fontan surgery and were in grades 1–3 (8–11 years of age) in 2023. After excluding children with a history of seizure, epilepsy, or brain infarction, 11 participants underwent standardized neurocognitive evaluation. Among them, four with extreme full-scale intelligence quotient (FSIQ) underwent 3T sedation-free ultrafast brain MRI (total scan time, 3 min 22 s), including volumetry-capable three-dimensional T1-weighted imaging. Six age-matched children served as controls. MRI volumetric analysis was exploratory and limited to a small subset of Fontan participants (*n* = 4), restricting statistical power and generalizability. Between-group comparisons were performed using Welch’s *t*-test, with Hedges’ g calculated as the effect size. **Results:** Mean FSIQ was 85.2 ± 24.3, with 36% patients with <85 FSIQ. Working memory (64%) and processing speed (55%) were most frequently impaired. Cerebellar volumes were lower in Fontan patients than in controls, although these differences were not statistically significant (left: 59.74 ± 8.86 vs. 72.26 ± 6.92 mL; right: 60.63 ± 7.70 vs. 71.54 ± 7.01 mL; very large effect sizes). Hippocampal volumes tended to be lower, and cerebellar volume showed a positive but non-significant correlation with processing speed. White matter hyperintensities and microbleeds were observed in two patients, both with impaired processing speed. **Conclusions:** School-aged Fontan patients exhibited selective deficits in working memory and processing speed, while exploratory MRI analysis suggested lower cerebellar volumes in the Fontan group. The ultrafast sedation-free MRI protocol proved feasible for volumetric assessment and, when combined with neurocognitive assessments, may support future milestone-based surveillance and early intervention for at-risk children.

## 1. Introduction

Advances in surgical and medical care have enabled the survival and growth to school age and adolescence of children with single-ventricle physiology. Despite improved hemodynamics following the Fontan operation, these patients remain at an increased risk of neurodevelopmental impairment possibly related to factors such as chronic hypoxemia, perioperative embolic insults, and repeated exposure to cardiopulmonary bypass [1]. Cognitive vulnerabilities often manifest as a lower intelligence quotient (IQ) score, executive function deficits, and academic difficulties that become more apparent with increasing developmental demands.

Recent multicenter studies have demonstrated a decline in neurocognitive scores from near-normal scores in infancy to below-average scores in school age, with IQs ranging from 85 to 90 and selective impairments in processing speed and working memory [2]. Structural brain abnormalities detected on magnetic resonance imaging (MRI), such as white matter hyperintensities and global or regional volume loss, have been associated with poor cognitive performance [3]. These findings underscore the need for a comprehensive longitudinal surveillance strategy that integrates neurodevelopmental analysis with neuroimaging and clinical neuromonitoring to guide early and timely interventions in at-risk children [4,5].

Nevertheless, data from Korean and other Asian populations are scarce. Specifically, no study has simultaneously reported neurocognitive outcomes and MRI-based volumetry in school-aged patients who have undergone Fontan operation in medium-volume centers. Furthermore, the need for sedation and lengthy scan times limits the use of MRI during routine follow-ups. Recent advances in sedation-free ultrafast brain MRI have demonstrated feasibility and clinically acceptable image quality, including multi-contrast three-dimensional (3D) acquisitions, such as NeuroMix, and two-dimensional (2D)-based pediatric fast protocols. These protocols can acquire multi-contrast images within minutes, including T1-weighted sequences suitable for volumetric analysis, thereby offering a practical opportunity to integrate neuroimaging into real-world clinical pathways in this vulnerable population [6,7,8,9].

This pilot study aimed to (1) evaluate neurocognitive profiles in early school-aged Fontan patients (grades 1–3; 8–11 years of age) using standardized analysis, (2) explore the feasibility of MRI volumetric analysis in a cognitively extreme subset, and (3) provide the early experience of using a rapid, sedation-free, ultrafast MRI protocol capable of volumetry, which may guide the development of a structured, regionally applicable neurodevelopmental follow-up program.

## 2. Methods

### 2.1. Study Population

In 2023, we screened children who had undergone Fontan surgery and were in grades 1–3 (8–11 years of age) for eligibility. Of the 25 screened children screened, 14 were excluded because of a history of seizures, epilepsy, brain infarction, or refusal to participate, leaving 11 eligible participants. These criteria were applied to reduce major neurologic confounding and to ensure cooperation with the sedation-free MRI protocol. All enrolled participants underwent standardized neurocognitive and clinical evaluations.

To explore the feasibility testing of MRI volumetry, four Fontan participants representing the extremes of full-scale IQ (FSIQ; two highest and two lowest) underwent sedation-free ultrafast brain MRI with deep learning-based reconstruction, including diffusion-weighted imaging (DWI), T2-weighted imaging (T2WI), T2-weighted fluid-attenuated inversion recovery (T2-FLAIR), three-dimensional echo planar imaging–susceptibility-weighted imaging (3D EPI-SWI), and 3D T1-weighted BRAVO imaging. This subgroup was selected to assess the practicality and motion tolerance with the sedation-free ultrafast MRI protocol across a broad cooperation spectrum and not to represent the broader Fontan population.

Six controls were selected from children who underwent normal brain MRI for headache evaluation during the same period using an exact- or nearest-age matching approach. Because recruitment of truly healthy volunteers for pediatric MRI is ethically and practically limited in a clinical setting, only children with normal structural MRI findings and no known neurological or developmental disorders were included as controls. Accordingly, the imaging analytical sample consisted of four Fontan participants and six age-matched controls.

**Figure 1:**
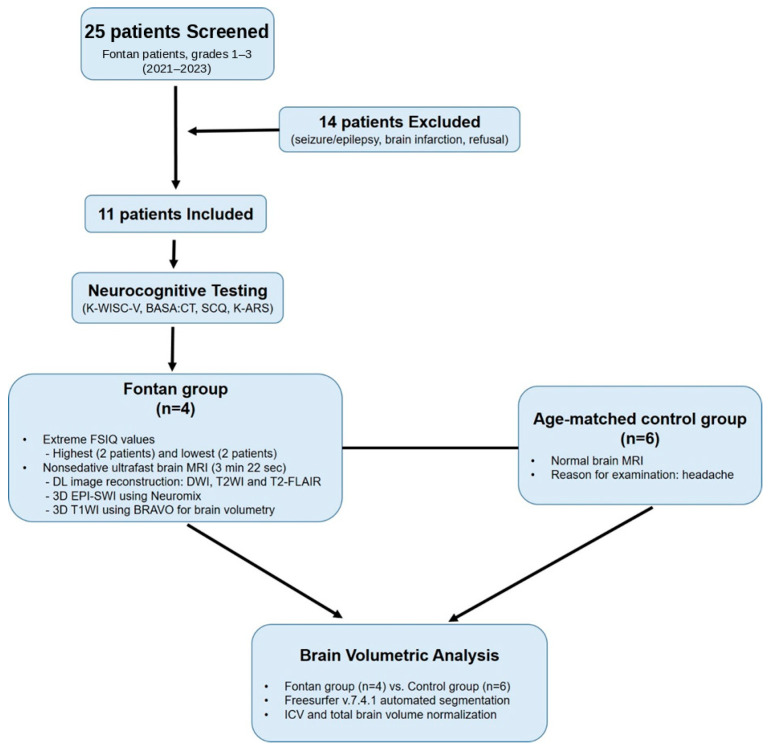
Study workflow. Study flowchart summarizing patient selection: 25 children were screened, 14 were excluded, 11 underwent neurocognitive testing, and 4 underwent brain MRI based on extreme FSIQ sampling; 6 age-matched controls with normal imaging findings were included for volumetric comparison. MRI, magnetic resonance imaging; FSIQ, full-scale intelligence quotient.

The flowchart of patient selection and group allocation is shown in Figure 1. The study protocol was approved by the Institutional Review Board of the Pusan National University Yangsan Hospital (IRB No. 05-2022-010) and complied with the Declaration of Helsinki [10]. Written parental informed consent and child assent were obtained.

### 2.2. Neurocognitive and Behavioral Assessment

All participants underwent standardized neurocognitive and behavioral evaluations. Cognitive function was evaluated using the Korean Wechsler Intelligence Scale for Children–Fifth Edition [11], which provides index scores for verbal comprehension, visual-spatial reasoning, fluid reasoning, working memory, and processing speed. Academic achievement was assessed using the Basic Academic Skills Assessment: Comprehensive Test (BASA:CT). Social communication ability was screened using the Social Communication Questionnaire (SCQ) [12], and attention-related symptoms were evaluated using the Korean Attention-deficit/hyperactivity Disorder Rating Scale (K-ARS) [13]. All composite scores were converted to age-standardized values based on national norms.

### 2.3. Sedation-Free Ultrafast Brain MRI Protocol, Including 3D T1-Weighted Image for Volumetry

MRI was performed on a 3 T scanner (Signa™ Architect; GE Healthcare, Waukesha, WI, USA) using a 48-channel head coil. The ultrafast protocol consists of optimized, accelerated versions of commercially available sequences, incorporating parallel imaging techniques to minimize scan time. The protocol included 3D T1-weighted imaging using the BRAVO sequence for volumetric analysis, axial T2WI, axial T2-FLAIR, DWI, and fast 3D EPI-SWI adapted from the NeuroMix sequence [14,15]. Of these, T2WI, T2-FLAIR, and DWI were reconstructed using a vendor-provided deep learning-based algorithm to improve image quality despite the shortened scan time. The total scan time was 3 min 22 s, and detailed sequence parameters are listed in Table 1.

### 2.4. Magnetic Resonance Volumetric Analyses and Post Hoc Analyses

Sagittal 3D T1-weighted images were analyzed using FreeSurfer (version 7.4.1; Harvard University, Boston, MA, USA), a software package for automated volumetric and surface-based brain segmentation [16]. All analyses were performed by a software engineer (JK). The software calculated the total intracranial volume; total brain volume excluding the ventricles; and hemisphere-specific volumes for the cortical gray matter, cerebral white matter, hippocampus, amygdala, caudate nucleus, putamen, globus pallidus, thalamus, and cerebellum.

As a control group, we included age-matched children with normal findings on brain MRI that were findings acquired during the same study period; their volumetric images were obtained using the same 3D T1-weighted BRAVO acquisition parameters and coil setup, and were processed using the same analysis pipeline. All analyses used the standard “recon-all” processing stream. After automated segmentation completion, a board-certified neuroradiologist (HJB) conducted a qualitative post hoc review to identify artifacts or segmentation-related errors in 3D T1-weighted images and discrepancies in color-coded outputs. All 3D T1-weighted images were visually inspected for motion artifacts by a board-certified neuroradiologist during post hoc quality-control review. No datasets were excluded because of motion, and residual artifacts were considered negligible for automated segmentation and volumetric analysis.

### 2.5. Statistical Analysis

Continuous variables were summarized as mean ± standard deviation. Given the small sample size, potential heteroscedasticity, and exploratory pilot design, between-group comparisons were performed using Welch’s *t*-test as a more robust alternative to the conventional Student’s *t*-test. Normality and variance tests were used as diagnostic assessments rather than as strict decision criteria. Effect sizes were calculated as Hedges’ g with small-sample correction and bias-corrected 95% confidence intervals (CIs). Correlations between cerebellar volume and neurocognitive scores were evaluated using Spearman’s rank correlation coefficients. Because of the exploratory pilot nature of the study, no multiple-comparison correction was applied; therefore, regional volumetric findings should be interpreted as hypothesis-generating. Exact *p* values, effect sizes, and 95% CIs were provided to aid interpretation. All analyses were performed in Python 3.11 using NumPy 1.26, SciPy 1.11 [17], pandas 2.1, statsmodels 0.14 and matplotlib 3.8.

## 3. Results

### 3.1. Patient Characteristics

Eleven children who had undergone Fontan procedure at a mean age of 3.5 ± 0.9 years were included (mean age at assessment, 8.1 ± 0.6 years; seven [64%] male). Among them, four patients with extreme FSIQ values and six age-matched children with normal brain MRI findings underwent MRI-based volumetry and served as the Fontan and control groups, respectively, resulting in 10 participants for volumetric comparison. The baseline demographic and clinical characteristics, including underlying cardiac diagnoses, are summarized in Table 2.

### 3.2. Neurocognitive and Behavioral Outcomes

The mean FSIQ was 85.2 ± 24.3, and four of 11 patients (36%) scored below 85. Among the cognitive domains, working memory and processing speed were the most frequently impaired (64% and 55%, respectively), both falling more than one standard deviation below the normative mean. The BASA:CT scores were generally within range for the corresponding grade, and the SCQ and K-ARS findings were unremarkable (Table 3).

### 3.3. MRI Findings Including Brain Volumetry

**Figure 2:**
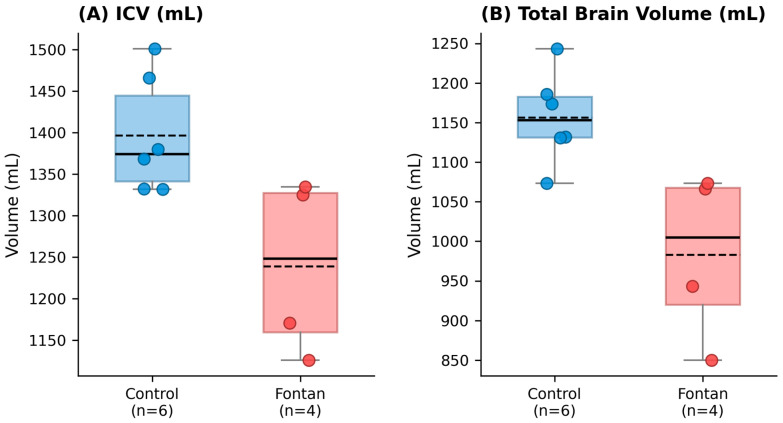
Box plots of global brain volumes in control participants and Fontan patients. (**A**) Total intracranial volume (ICV) and (**B**) total brain parenchymal volume (excluding ventricles). Boxes represent the interquartile range, whiskers indicate the range, horizontal lines represent the median, and dashed lines represent the group mean. Individual data points are overlaid to illustrate the underlying distribution given the small sample size (Control, *n* = 6; Fontan, *n* = 4). Exact *p* values and detailed statistical results are provided in Table 4. ICV, intracranial volume.

Table 4 summarizes the volumetric measurements derived from the fast 3D T1-weighted sequences processed using FreeSurfer. Fontan patients showed lower mean intracranial volume than controls (1239.04 ± 106.53 mL vs. 1396.64 ± 70.97 mL; *p* = 0.050), with a large standardized mean difference (Hedges’ g = 1.65 [95% CI, 0.15–3.16]). The mean total brain parenchymal volume excluding the ventricles was also lower in the Fontan group than in the control group (983.12 ± 107.07 mL vs. 1156.42 ± 58.11 mL; *p* = 0.039), with a very large effect size (Hedges’g = 1.96 [95% CI, 0.37–3.54]). The global volume distribution is illustrated in Figure 2.

**Figure 3:**
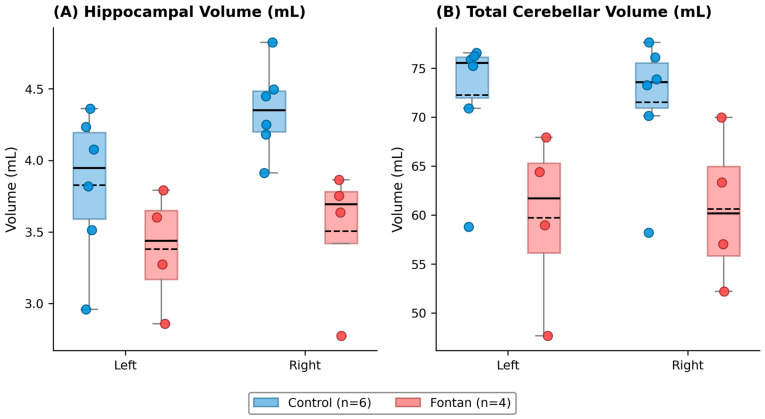
Box plots of regional brain volumes in control participants and Fontan patients. (**A**) Left and right hippocampal volumes and (**B**) left and right total cerebellar volumes (cortex + white matter). Boxes represent the interquartile range, whiskers indicate the range, horizontal lines represent the median, and dashed lines represent the group mean. Individual data points are overlaid to illustrate the underlying distribution given the small sample size (Control, *n* = 6; Fontan, *n* = 4). Exact *p* values and detailed statistical results are provided in Table 5.

Regional volumetric comparisons are summarized in Table 5 and Figure 3. Lower mean volumes were observed in Fontan patients across several structures, including cortical gray matter, cerebral white matter, hippocampus, amygdala, caudate nucleus, and cerebellum. Although some between-group differences appeared more prominent in right-sided structures, these laterality patterns should be interpreted cautiously given the very small imaging sample.

**Figure 4:**
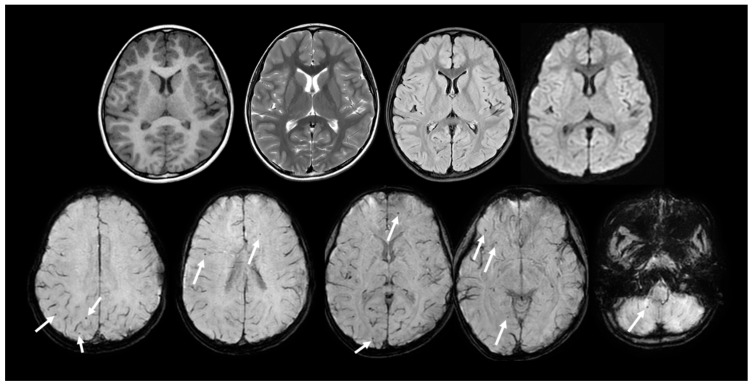
Representative structural findings on sedation-free ultrafast brain MRI in Fontan patients. Upper row: Representative sedation-free ultrafast brain MRI sequences (T1-weighted image, T2-weighted image, FLAIR and DWI) from a Fontan patient without significant structural abnormalities. Lower row: Susceptibility-weighted imaging from the ultrafast protocol demonstrating multiple small hypointense foci (arrows), consistent with cerebral and cerebellar microbleeds. These findings exemplify subclinical microvascular injury that may contribute to cognitive vulnerability in this population. MRI, magnetic resonance imaging; FLAIR, fluid-attenuated inversion recovery; DWI, diffusion-weighted image.

Among the volumetric findings, total cerebellar volume showed a positive correlation with the processing speed index (Spearman ρ = 0.85). However, this association was not statistically significant. In addition, scattered white matter hyperintensities and several microbleeds were observed in two Fontan patients, both exhibiting impaired processing speed on sedation-free ultrafast brain MRI (Figure 4).

### 3.4. Discussion

This pilot study evaluated the neurodevelopmental status of early school-aged children with single-ventricle physiology following the Fontan operation, and explored the feasibility of brain MRI volumetry in follow-up care. The mean FSIQ score in our cohort was in the lower average range, with working memory and processing speed deficits emerging as the most vulnerable domains, in keeping with prior studies reporting selective cognitive vulnerability in Fontan survivors [2,18,19]. Although the BASA:CT scores performance and screening results for autism spectrum disorder and attention-deficit/hyperactivity disorder were generally unremarkable, these domain-specific weaknesses may still affect daily classroom functioning and social adaptation during the early school years.

Our exploratory MRI analysis suggested lower global and regional brain volumes in the Fontan group. Compared with controls, Fontan patients showed lower mean intracranial and total brain parenchymal volumes, as well as lower mean regional volumes in the hippocampus, white matter, amygdala, caudate nucleus, and cerebellum. However, given the very small imaging sample, extreme-case FSIQ sampling, and exploratory design, these imaging findings should be interpreted as preliminary and hypothesis-generating rather than confirmatory. Prior studies have reported a similar cerebellar, hippocampal, and broader structural brain vulnerability in Fontan and congenital heart disease cohorts [18,19,20,21]. In addition, some between-group differences appeared more prominent in right-sided structures in our data, but these laterality patterns should be interpreted cautiously and may reflect random variation in a very small sample.

Structural brain lesions, including white matter hyperintensities and microbleeds, were observed in a subset of patients. These findings may reflect subclinical cerebrovascular vulnerability relevant to neurocognitive outcome, consistent with prior reports linking white matter abnormalities to reduced processing speed and describing microbleeds after cardiopulmonary bypass in children [3,22]. An important finding of the present study is the practical feasibility of sedation-free ultrafast brain MRI in this population. In our cohort, multi-contrast MRI including volumetry-capable 3D T1-weighted imaging was completed within 3 min 22 s without sedation, and all examinations were completed without repeat acquisition. Prior ultrafast MRI studies have shown that abbreviated pediatric brain MRI protocols can provide acceptable image quality with reduced motion burden in routine practice [6,7,8,9,14,15]. In this context, our findings support the feasibility of applying such an approach to Fontan follow-up. Broader developmental imaging studies in congenital heart disease have also shown progressive structural abnormalities from early life through school age, supporting the rationale for continued neurodevelopmental monitoring in this population [3,19,21,23,24,25].

This study has several limitations inherent to its pilot design. The MRI volumetric analysis was exploratory and based on a very small sample, and the use of an extreme-case FSIQ strategy may have introduced selection bias and limited generalizability. Recruitment for paired neurocognitive testing and MRI was also challenging in this clinical setting, and, together with the limited number of long-term Fontan survivors available at a single institution, this contributed to the feasibility-oriented design of the study. The control group consisted of children undergoing MRI for headache evaluation rather than healthy volunteers, which may have introduced residual confounding, although all controls had normal structural MRI findings and no known neurological or developmental disorders. In addition, this was a single-center cross-sectional pilot study, and no multiple-comparison correction was applied; therefore, all regional volumetric findings should be regarded as exploratory. Although prior research has supported the clinical applicability of ultrafast MRI in pediatric practice [6,8,26], the present study does not establish longitudinal sensitivity or reproducibility. Moreover, while international recommendations support neurodevelopmental follow-up in congenital heart disease, implementation remains heterogeneous across centers [1,27,28]. These limitations, together with the current heterogeneity of follow-up strategies across centers, support the need for future prospective multicenter studies using harmonized surveillance protocols.

## 4. Conclusions

In conclusion, this pilot study demonstrates the feasibility of combining neurocognitive testing with sedation-free ultrafast brain MRI in a selected subgroup of school-aged Fontan patients. This approach may have potential utility for future neurodevelopmental monitoring, but its broader clinical role and longitudinal applicability remain to be established. Larger prospective multicenter studies with harmonized surveillance protocols are warranted to validate these preliminary observations and to define the clinical role of imaging-integrated neurodevelopmental follow-up in Fontan survivors.

## Figures and Tables

**Table 1:** Representative acquisition parameters for pediatric ultrafast MRI.

Imaging Parameter	Ultrafast MRI				
	3D T1WI *	Axial T2WI ^†^	Axial T2-FLAIR ^†^	Axial DWI ^†^	Fast-SWI
FOV (mm)	240	220	220	240	230
Section thickness (mm)	1	5	5	5	5
TR (ms)	6.5	3000	6675	2850	44
TI (ms)	-	-	2150	-	-
TE (ms)	2.5	100	106	75	20
ETL	-	16	21	-	8
Frequency matrix	240	416	330	128	300
Phase matrix	240	256	224	128	200
Flip angle (°)	12	142 ^‡^	160 ^†^	90	18
Bandwidth (±kHz/FOV)	41.67	62.5	62.5	250	111.11
Parallel imaging acceleration factor	ARC:2, HS:1.2	ARC:2.5	ARC:2.5	ARC:2, HB:2	ARC:2
Net scan time (min:s)	1:29	0:27	1:00	0:14	0:12
Total scan time (min:s)	3:22				

Note.—* 3D T1-weighted volumetric images were obtained with a BRAVO sequence. ^†^ Sequences were reconstructed using a vendor-provided deep learning-based reconstruction algorithm to reduce noise and improve image quality. The SWI sequence used in this study was a standalone fast 3D EPI-SWI (kSEPI2) sequence (12 s), inspired by the NeuroMix concept. NeuroMix is a 1-min multi-contrast brain MRI sequence that combines EPI and FSE readouts, incorporating both 2D and 3D components within a single acquisition. ^‡^ indicates refocusing flip angle. ARC, autocalibrating reconstruction for Cartesian imaging; DWI, diffusion-weighted imaging; ETL, echo train length; NeuroMix, a 1-min multi-contrast brain MRI sequence using segmented echo-planar imaging; FLAIR, fluid-attenuated inversion recovery; FOV, field of view; HB, HyperBand; HS, HyperSense; SWI, susceptibility-weighted imaging; T1WI, T1-weighted imaging; T2WI, T2-weighted imaging; TE, echo time; TI, inversion time; TR, repetition time.

**Table 2:** Baseline characteristics of the study cohort.

Characteristic	Value
**Age at assessment (years)**	8.1 ± 0.6
**Male sex, n (%)**	7 (64)
**Age at Fontan completion (years)**	3.5 ± 0.9
**Cardiac Diagnoses**	
Hypoplastic left heart syndrome	2
Tricuspid atresia	3
Double-outlet right ventricle (±LV hypoplasia, ccTGA)	2
Pulmonary atresia with intact ventricular septum	1
DILV	1
TGA/VSD with pulmonary stenosis	1
VSD with TV straddling	1

Note.—Values are presented as mean ± standard deviation or number of patients (%). LV = left ventricle; ccTGA = congenitally corrected transposition of the great arteries; DILV = double-inlet left ventricle; TGA = transposition of the great arteries; VSD = ventricular septal defect; TV = tricuspid valve.

**Table 3:** Neurocognitive and behavioral results.

Measure	Mean ± SD	n (%) < 85 or Abnormal
**K-WISC-V**		
Full-Scale IQ (FSIQ)	85.2 ± 24.3	4 (36)
Verbal Comprehension	84.4 ± 32.8	4 (36)
Visual Spatial	88.1 ± 18.5	5 (45)
Fluid Reasoning	93.0 ± 22.2	4 (36)
Working Memory	80.5 ± 14.2	7 (64)
Processing Speed	84.8 ± 16.4	6 (55)
**BASA:CT**	Within grade level	0 (0)
**SCQ**	Within normal range	0 (0)
**K-ARS**	Within normal range	0 (0)

Note.—Values are mean ± standard deviation unless otherwise noted. Percentages indicate the number of patients with scores below 85 (K-WISC-V) or outside the normal range (BASA:CT, SCQ, K-ARS).

**Table 4:** Comparison of total intracranial and total brain volumes between controls and Fontan patients using FreeSurfer.

	Normal Control (Mean ± SD)	Fontan Patients (Mean ± SD)	*p*	Hedges’ g [95% CI]
ICV	1396.64 ± 70.97	1239.04 ± 106.53	0.050	1.65 [0.15, 3.16]
Total brain volume *	1156.42 ± 58.11	983.12 ± 107.07	0.039	1.96 [0.37, 3.54]

Note.—The units are mL. Values are mean ± standard deviation (SD). CI= confidence interval, ICV= total intracranial volume. * means total brain volume without ventricles. *p* values were calculated using Welch’s *t*-test (assuming unequal variances) for comparisons between Fontan patients and controls. Effect sizes are presented as Hedges’ g with 95% CIs.

**Table 5:** Comparison of cerebral and cerebellar hemispheric volumes between age-matched controls and Fontan patients using FreeSurfer.

Variables	Left			Right		
	Normal Control (Mean ± SD)	Fontan Patients (Mean ± SD)	*p* (Hedges’ g, 95% CI)	Normal Control (Mean ± SD)	Fontan Patients (Mean ± SD)	*p* (Hedges’ g, 95% CI)
Cortical gray matter	279.11 ± 14.81	239.89 ± 32.84	0.091 1.52 [0.05, 2.99]	285.12 ± 12.14	238.70 ± 32.10	0.057 1.92 [0.34, 3.49]
Cerebral white matter	192.80 ± 19.98	162.27 ± 21.52	0.063 1.34 [−0.09, 2.77]	195.45 ± 17.94	163.34 ± 17.20	0.026 1.64 [0.14, 3.14]
Hippocampus	3.83 ± 0.52	3.38 ± 0.41	0.171 0.83 [−0.49, 2.16]	4.35 ± 0.31	3.51 ± 0.50	0.033 1.95 [0.36, 3.53]
Amygdala	1.58 ± 0.16	1.44 ± 0.19	0.269 0.74 [−0.57, 2.06]	1.74 ± 0.13	1.47 ± 0.09	0.005 2.08 [0.45, 3.70]
Caudate	3.84 ± 0.53	3.26 ± 0.24	0.052 1.16 [−0.23, 2.55]	4.06 ± 0.51	3.31 ± 0.24	0.015 1.58 [0.10, 3.06]
Putamen	5.69 ± 0.89	4.71 ± 1.26	0.238 0.84 [−0.49, 2.17]	5.92 ± 0.75	5.16 ± 1.11	0.288 0.76 [−0.56, 2.07]
Pallidum	2.16 ± 0.25	1.98 ± 0.36	0.434 0.54 [−0.75, 1.83]	2.17 ± 0.25	2.48 ± 0.75	0.478 −0.56 [−1.85, 0.74]
Thalamus	8.58 ± 0.88	7.35 ± 1.17	0.131 1.11 [−0.26, 2.49]	8.39 ± 0.93	7.67 ± 0.90	0.267 0.70 [−0.61, 2.01]
Cerebellum	72.26 ± 6.92	59.74 ± 8.86	0.059 1.47 [0.01, 2.92]	71.54 ± 7.01	60.63 ± 7.70	0.063 1.35 [−0.07, 2.78]

Note.—The units are mL. Values are mean ± standard deviation (SD). *p* values were calculated using Welch’s *t*-test (assuming unequal variances) for comparisons between Fontan patients and controls. Effect sizes are presented as Hedges’ g with small-sample correction and 95% confidence intervals (CIs). Given the exploratory pilot design and small sample size, these regional volumetric findings should be interpreted as exploratory and hypothesis-generating.

## Data Availability

The data that support the findings of this study are available from the corresponding author upon reasonable request. Individual participant data are not publicly available due to privacy and ethical restrictions.

## Abbreviations

ARC	autocalibrating reconstruction for Cartesian imaging;
BASA:CT	Basic Academic Skills Assessment: Comprehensive Test;
CI	confidence interval;
DWI	diffusion-weighted imaging;
EPI	echo-planar imaging;
FLAIR	fluid-attenuated inversion recovery;
FSIQ	full-scale intelligence quotient;
ICV	intracranial volume;
IRB	Institutional Review Board;
K-ARS	Korean ADHD Rating Scale;
K-WISC-V	Korean Wechsler Intelligence Scale for Children–Fifth Edition;
MRI	magnetic resonance imaging;
SWI	susceptibility-weighted imaging;
T1WI/T2WI	T1-/T2-weighted imaging;
TGA	transposition of the great arteries;
VSD	ventricular septal defect.
